# Biocrude Production Using a Novel Cyanobacterium: Pilot-Scale Cultivation and Lipid Extraction via Hydrothermal Liquefaction

**DOI:** 10.3390/su15064878

**Published:** 2023-03-09

**Authors:** Samson Gichuki, Behnam Tabatabai, Viji Sitther

**Affiliations:** 1 Department of Biology, Morgan State University, 1700 E. Cold Spring Lane, Baltimore, MD 21251, USA; 2 HaloCyTech LLC, 4709 Harford Road, Baltimore, MD 21214, USA

**Keywords:** biochar, biocrude, fatty acids, *Fremyella diplosiphon*, photosynthetic pigments, scale-up optimization

## Abstract

The use of renewable energy to reduce fossil fuel consumption is a key strategy to mitigate pollution and climate change, resulting in the growing demand for new sources. Fast-growing proprietary cyanobacterial strains of *Fremyella diplosiphon* with an average life cycle of 7–10 days, and a proven capacity to generate lipids for biofuel production are currently being studied. In this study, we investigated the growth and photosynthetic pigmentation of a cyanobacterial strain (SF33) in both greenhouse and outdoor bioreactors, and produced biocrude via hydrothermal liquefaction. The cultivation of *F. diplosiphon* did not significantly differ under suboptimal conditions (*p* < 0.05), including in outdoor bioreactors with growth differences of less than 0.04 (*p* = 0.035) among various batches. An analysis of the biocrude’s components revealed the presence of fatty acid biodiesel precursors such as palmitic acid and behenic acid, and alkanes such as hexadecane and heptadecane, used as biofuel additives. In addition, the quantification of value-added photosynthetic pigments revealed chlorophyll *a* and phycocyanin concentrations of 0.0011 ± 5.83 × 10^−5^ μg/μL and 7.051 ± 0.067 μg/μg chlorophyll *a*. Our results suggest the potential of *F. diplosiphon* as a robust species that can grow at varying temperatures ranging from 13 °C to 32 °C, while producing compounds for applications ranging from biofuel to nutritional supplements. The outcomes of this study pave the way for production-level scale-up and processing of *F. diplosiphon*-derived biofuels and marketable bioproducts. Fuel produced using this technology will be eco-friendly and cost-effective, and will make full use of the geographical location of regions with access to brackish waters.

## 1. Introduction

Toxic air pollutants and carbon dioxide released into the atmosphere due to fossil fuel combustion are major threats to the environment, leading to climate change, and have devastating effects on the planet [1]. In addition, exposure to the diesel exhaust produced by these fuels is linked to respiratory symptoms, lung inflammation, and other chronic diseases, especially in children and the immuno-compromised elderly population [2,3]. The most common fossil fuels, such as oil, natural gas, and coal, generate 85% of airborne particulate pollution and nearly all of the sulfur dioxide and nitrogen oxide released into the atmosphere [4]. Thus, the overuse of fossil fuels has adverse effects on the environment, which has led to an increase in the demand for renewable energy in recent years [5]. It is more important than ever to find alternative sources of energy that do not compete with food crops, and are sustainable, environmentally friendly, and cost-effective.

Biofuels are potential alternatives to fossil fuels since they are produced through processes that significantly reduce net carbon emissions [6]. However, while several initiatives aim to reduce emissions by limiting fossil fuel consumption, the use of biofuels as a renewable energy source has not increased significantly due to high capital and cultivation costs. A major constraint that limits the economic viability of cyanobacterial biofuel production is the high capital cost involved in large-scale cultivation of these organisms, thus driving the need for optimized cultivation systems. Since cost-efficiency is a major concern for fuel producers, distributors, and consumers transitioning from fossil fuels to biofuels, this technology may offer the potential to transform the energy industry only if the capital expenditure and cultivation costs can be reduced. While great opportunities exist to increase the use of renewable fuels, it is important that biofuels provide a net energy gain and economic competitiveness for enhanced sustainability [7]. Producing biodiesel from algae/cyanobacteria demands the use of efficient strains, as well. Until these options become available, the use of cyanobacterial-derived biofuel will not be a feasible option, despite being the best choice for the health of planet Earth. Looking to the future, it is estimated that water and land resources in the U.S. could support the production of 23.5 billion gallons/year of algae-based fuel [8]. In addition, the cost of fuel production could be reduced if brackish/saline waters is utilized effectively. This technology may then greatly benefit island nations where fuel import costs are high and freshwater is too precious for use in cyanobacterial/algal growth, but where abundant saltwater exists.

Cyanobacteria produce about 50% lipids in their cells to store energy, which is extracted and used as biodiesel via transesterification. A theoretical oil yield of 38,000 gallons/acre/year and a practical yield of 4350–5700 gallons/acre/year are reported from cyanobacteria/algae [9]. With a fast generation time of 10 days, high biomass conversion, greenhouse gas fixation ability, and the capacity to produce lipids, cyanobacteria are viable platforms for biofuel production. Concentrated carbon dioxide released from fossil fuels and industrial emissions is efficiently captured by these organisms and used in the process of photosynthesis.

Currently, the most common approach to convert cyanobacterial lipids into biofuels is transesterification [10,11]. In addition to this method, hydrothermal liquefaction is another process that can yield biofuels even from typically recalcitrant organisms with low extractable lipids and high moisture content [12]. However, instead of biodiesel, the primary product of this reaction is biocrude. This product is more efficient for use in different types of fuels as it can be incorporated in a conventional refinery to be mixed with crude oil. The efficiency of the process has been previously demonstrated in the microalgae *Chlorella* and cyanobacteria *Spirulina* [13]. An additional benefit of this process is the ability to couple bioenergy production with the recycling and remediation of wastewater [14].

The filamentous cyanobacterium *Fremyella diplosiphon* is a preferred model organism since it has a short life cycle of 7–10 days, requires low light intensity for optimal growth, and can be manipulated with ease. A total lipid content of 18% has been reported in this cyanobacterium, which is capable of being converted to fatty acid methyl esters (FAMEs), making it an ideal biodiesel agent [11]. However, the viability of this organism as a commercial-scale feedstock with regards to cultivation and extraction has not been reported. In the present study, we evaluated the production process and identified technical obstacles associated with scaled-up cultivation of *F. diplosiphon* in greenhouse and outdoor conditions using naturally available brackish waters (5–20% NaCl). After modifying and optimizing bioreactor conditions, we evaluated culture growth and photosynthetic pigment accumulation. In addition, we performed hydrothermal liquefaction and assessed the resultant biocrude and other co-products, including biochar. We report a pathway to further develop the technology toward market-scale production.

## 2. Methods

### 2.1. Strain and Culture Conditions

*F. diplosiphon* strain SF33, a short filamentous strain, used in this study was obtained from Dr. Beronda Montgomery at Michigan State University (East Lansing, MI, USA). Seed cultures at the laboratory scale were grown from Petri plates containing BG-11 cyanobacterial medium [15] supplemented with 20 mM HEPES buffer. Cultures were then transferred to liquid BG-11 in flasks under continuous shaking at 170 rpm and 28 °C for 7 days. Cultures initiated in the laboratory were transferred to pilot settings using inoculation in 10-gallon fish tanks or 20 L bioreactors in a greenhouse at Morgan State University, Baltimore, MD. We maintained three bioreactors that served as biological replicates, and the mean of three technical replicates from each bioreactor was calculated. In addition, cultures in both the greenhouse and scale-up experiments were observed every six days under a light microscope (Motic, Schertz, TX, USA) to detect contaminants or morphological alterations.

### 2.2. Quantification of Pigments

Phycocyanin and chlorophyll *a* fluorescence in *F. diplosiphon* were recorded every other day using a BioTek Synergy H1 Microplate Reader (Agilent, Santa Clara, CA, USA). Chlorophyll *a* fluorescence was recorded at an excitation of 420 nm and an emission of 680 nm, and phycocyanin at an excitation of 590 nm and an emission of 650 nm [16]. Pigment levels were quantified at the initiation and completion of this study. Additionally, chlorophyll *a* and phycocyanin were extracted and quantified as previously described in [17,18] to determine the cellular photosynthetic efficiency. Phycobiliprotein levels were calculated according to the procedure described in [18] and reported relative to chlorophyll *a* as described in [19].

### 2.3. Optimization of Indoor Reactor Systems

Cultures were grown in 10-gallon aquarium tanks under greenhouse conditions using brackish waters collected from the Morgan State Patuxent Environmental Aquatic Research Laboratory in Calvert County, MD (38.39, −76.51). Water from the Patuxent River in the Chesapeake Watershed was filtered, followed by UV treatment, and 5 L was used for growth studies in 10-gallon fish tanks. The OD_750_ of the cultures was adjusted to 0.1 and the cultures were aerated using standard 10-gallon aquarium air pumps. Design parameters were modified and optimized as needed to mitigate technical risks and increase biomass yield. OD_750_ was measured every three days over the course of the experiment.

Cultivation of *F. diplosiphon* under greenhouse conditions was scaled-up to 5 L, 10 L, and 15 L cultures in 20 L Nalgene carboys (Thermo Fisher, Waltham, MA, USA). A novel bioreactor design was devised to agitate the cultures for gaseous exchange. Growth as a measure of OD_750_ was measured every three days over the lifespan of the cultures. The temperature and humidity in the greenhouse were monitored using a data logger (Elitech, San Jose, CA, USA). Growth between the bioreactors was compared to determine cultivation consistency.

Cultivation of *F. diplosiphon* in bioreactors was performed as mentioned above under outdoor conditions in Baltimore County, MD, USA (39.38, −76.517) in July 2022. Daily temperature was recorded using a data logger as mentioned previously.

The mean growth (OD_750_) and pigmentation were calculated and the statistical significance determined using one-way analysis of variance (ANOVA) and Tukey’s honest significant differences post hoc test at 95% confidence intervals (*p* < 0.05) (Supplementary Materials Tables S1 and S2). The single factor, fixed-effect ANOVA model, Yij = μ + αGi + εij, was used where Y is the growth or pigmentation in strain i and technical replicate j, μ represents mean growth or pigmentation with adjustments from effects of strain (αG), and εij is the experimental error from strain i and technical replicate j.

#### Hydrothermal Liquefaction and Biocrude Extraction:

Cultures were allowed to settle overnight by cordoning off aeration, and the biomass was collected the following day. Hydrothermal liquefaction was performed in a 100 mL Hydrothermal Synthesis Autoclave Reactor (6 Mpa, 240 °C, 304 stainless steel, high pressure) with polytetrafluoroethylene lining acid and an alkali resistance reactor (Baoshishan, China), loaded with 50 mL wet biomass and 30 mL 1M acetic acid as an acid catalyst, using a modified version of a protocol described previously [13]. The reactor was placed in a Lindberg/Blue M Box Furnace commercial oven (ThermoFisher, Waltham, MA, USA) and heated at 220 °C for 3, 6, or 16 h. Following hydrothermal liquefaction, a 1:1 mixture of dichloromethane (DCM): water was added to the reaction mixture for phase separation. The reaction mixture was decanted to recover the solid biochar, and the DCM phase was evaporated using a rotary evaporator (Heidolph, Wood Dale, IL, USA) to extract biocrude.

#### Gas chromatography–mass spectrometry (GC–MS):

The composition of the biocrude oil was analyzed using gas chromatography-mass spectrometry (Agilent Technologies, Santa Clara, CA, USA) using a HP5-MS capillary column (30 m, 0.25 mm id, 0.25 mm film thickness). The inlet temperature and split ratio were maintained at 300 °C and 20:1, respectively. The sample (2 μL) was then injected into the GC–MS system consisting of an Agilent 7890B gas chromatograph and Agilent 5977B mass selective detector (Agilent, USA). The temperature of the column was initially held at 50 °C for 5 min and then ramped up to 300 °C at a rate of 10 °C min^−1^. Upon attaining 300 °C, the temperature was maintained isothermally for 4 min, thereby amounting to a total run time of 37 min. Helium was used as the carrier gas with a constant flow rate of 1.6 mL min^−1^. Data acquisition of the chromatogram peaks was carried out using the MassHunter WorkStation, and the probable compounds were identified by conducting similarity analysis using the National Institute of Standards and Technology (NIST) Mass Spectral Library database. GC-MS was carried out at the Mass Spectrometry Core Facility at Johns Hopkins University (Baltimore, MD, USA). Lastly, Fourier transform-infrared (FT-IR) spectra of *F. diplosiphon* biomass, biochar, and biocrude were recorded using an IRSpirit (QATR-S) spectrophotometer (Shimadzu Corp., Kyoto, Japan) and their respective structures compared.

## 3. Results

### Cultivation studies:

**Figure 1.**
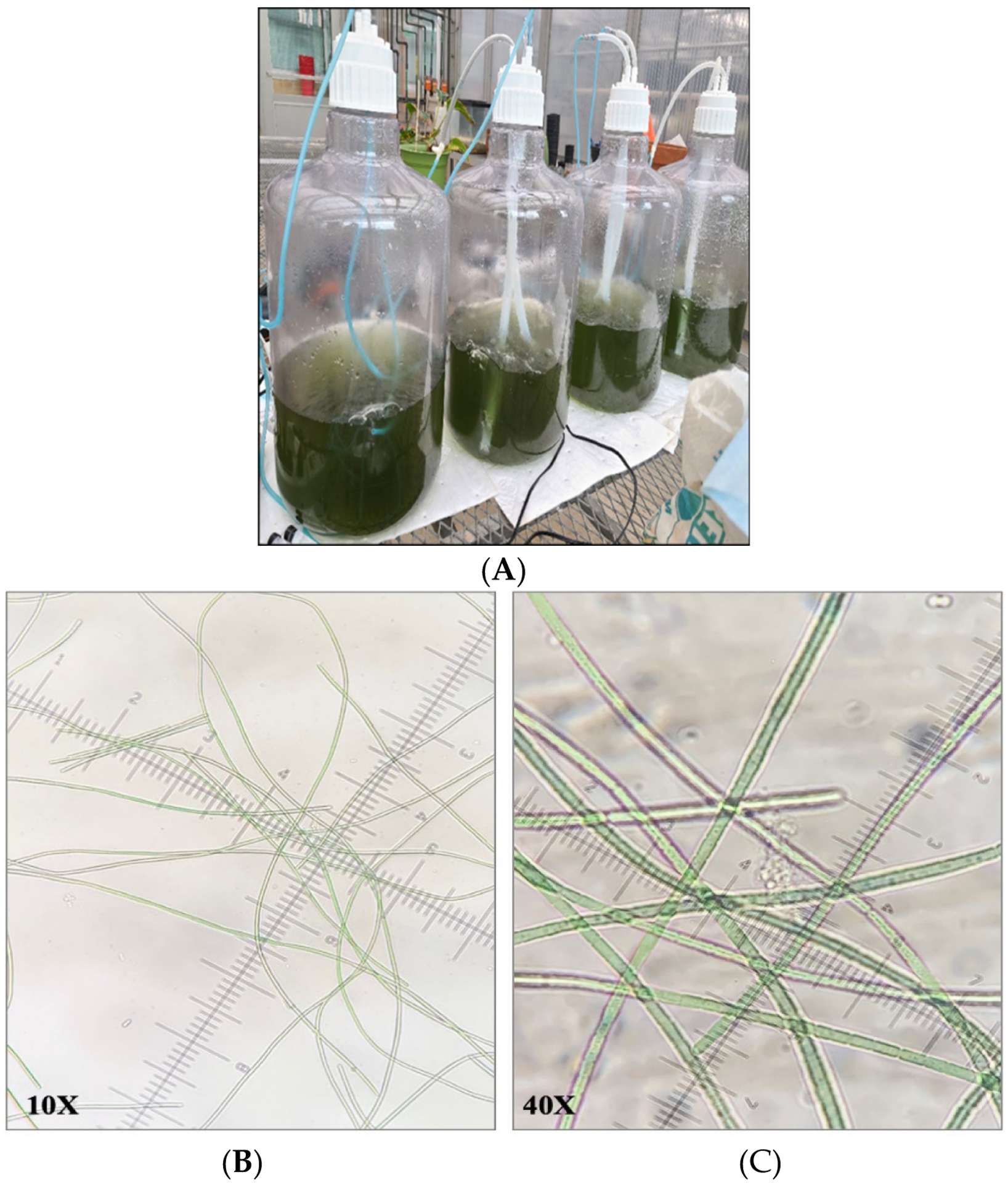
(**A**) 20 L carboys used as bioreactors in this study. Observation under a microscope at 10× (**B**) and 40× (**C**) magnification confirmed the absence of contaminants in the cultures of *Fremyella diplosiphon* grown in bioreactors.

**Figure 2.**
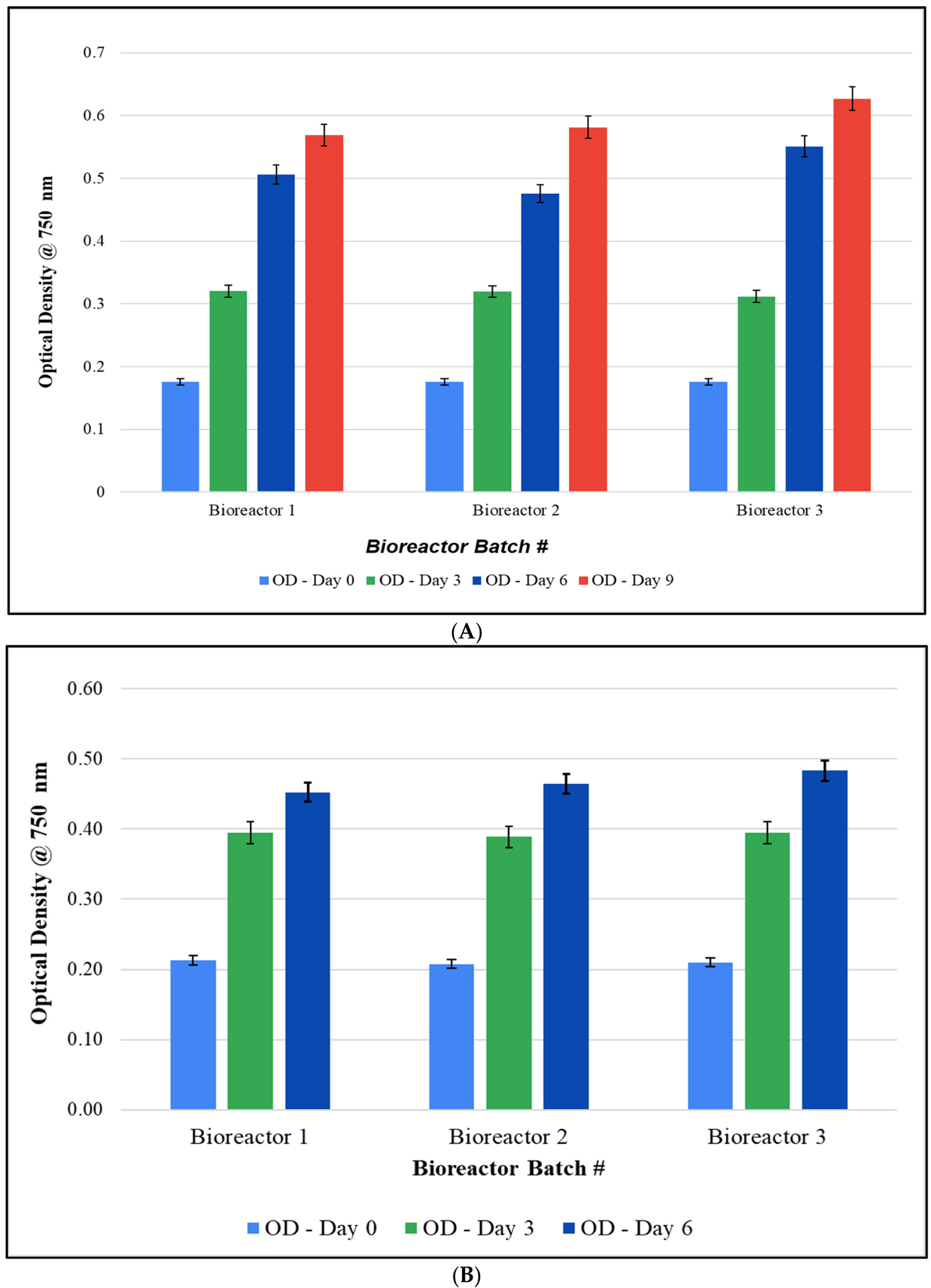

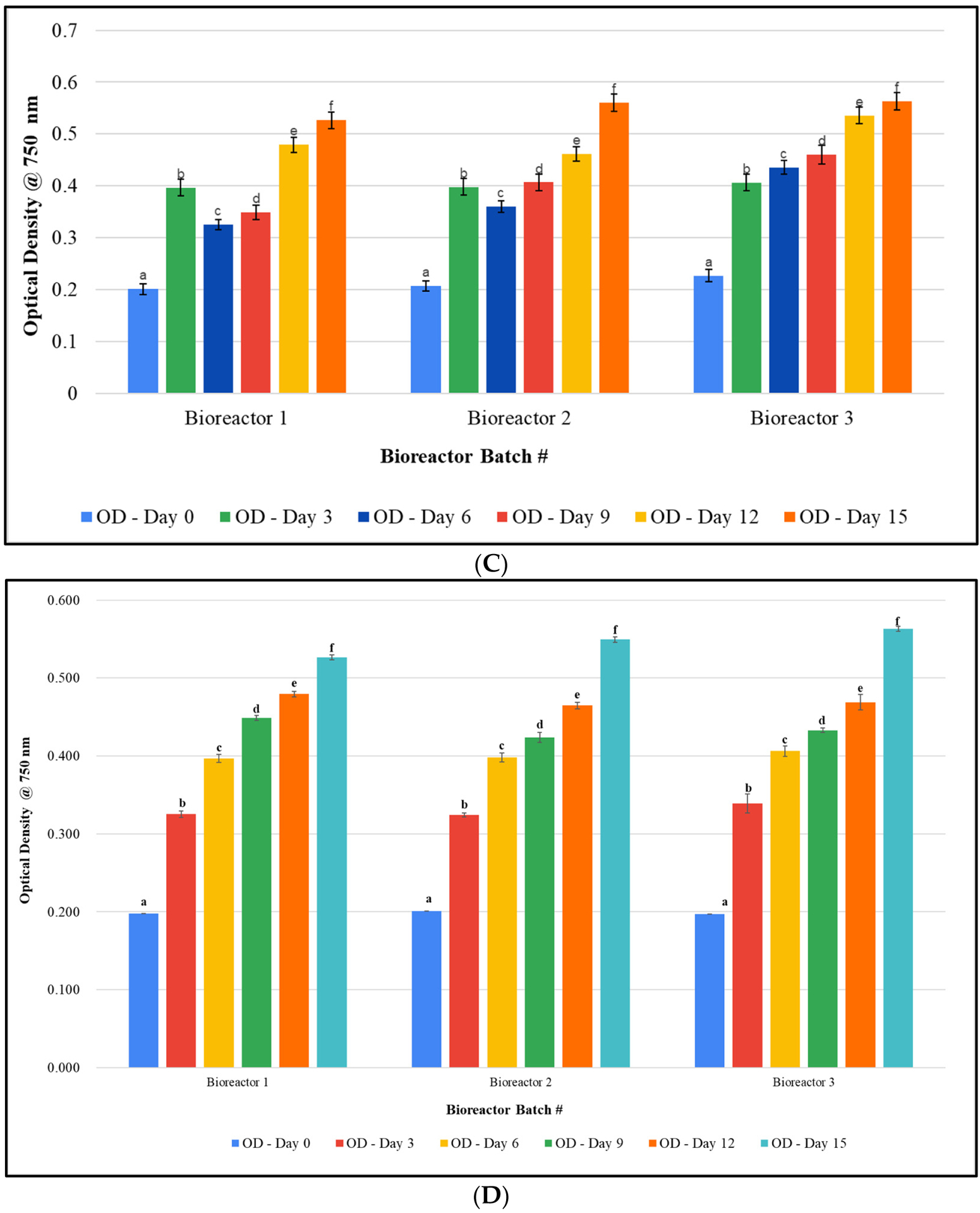
Growth of *Fremyella diplosiphon* SF-33 in 20 L bioreactors: (**A**) 5 L, (**B**) 10 L, and (**C**) 15 L cultures in the greenhouse and (**D**) 15 L outdoors. The average optical density at 750 nm (±standard error) of three technical replicates for each bioreactor over a 15-day period is shown. Identical letters above bars indicate no significant difference among bioreactor means on a given day (*p* < 0.05).

Since studies in 10-gallon aquariums resulted in suboptimal growth of *F. diplosiphon*, we implemented a series of modifications to improve cultivation. The use of 20 L bioreactors mitigated the culture loss from the evaporation observed in fish tank cultivation (Figure 1A). In addition, the use of 3/16^*”*^ airline tubing enhanced aeration by enabling larger bubbles and improving circulation. This also prevented the stagnation and settling of cultures, and as a result, growth was significantly increased. In addition, closing the open holes in the bioreactor cap prevented water loss due to evaporation. We also observed that an initial OD_750_ of 0.2 instead of 0.1 enabled rapid establishment of the culture. As a result, observation under a light microscope revealed the absence of contaminants (Figure 1B,C). Cultures grown both in the greenhouse (5 L, 10 L, and 15 L) and in outdoor (15 L) conditions demonstrated the ability of the strain to grow under fluctuating temperatures ranging from 13 to 32 °C (Figure 2). In addition, scaled-up cultivation did not significantly differ between batches at all volumes and conditions (*p* < 0.05). After 15 days, the OD_750_ of the three outdoor bioreactors varied by less than 0.04, with values of 0.527 ± 0.055, 0.549 ± 0.059, and 0.563 ± 0.053 for bioreactors 1, 2, and 3, respectively (*p* = 0.035).

### Pigments quantified in scale-up cultures:

**Figure 3.**
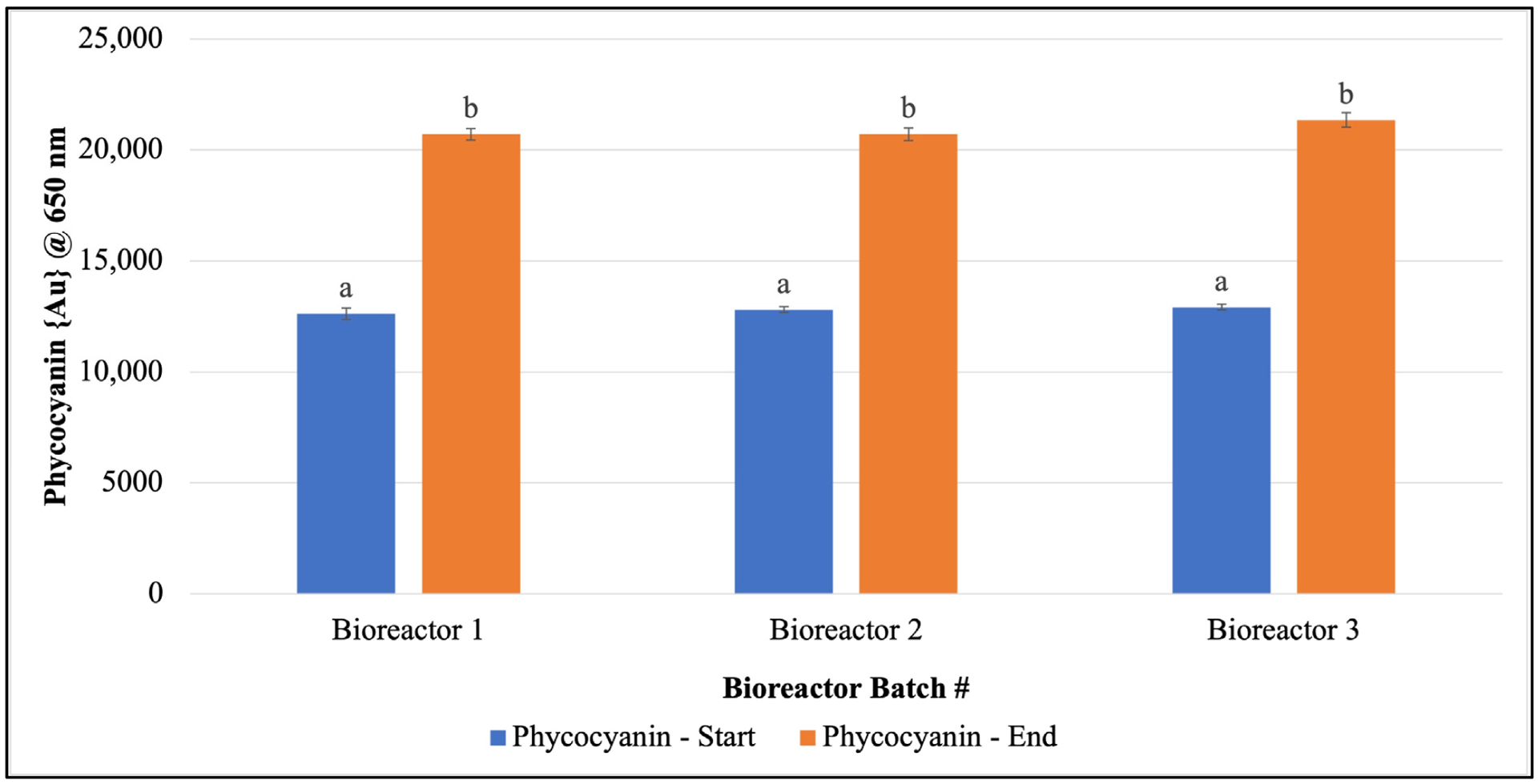
Increase in *Fremyella diplosiphon* phycocyanin content over a 15-day period in 15 L bioreactor cultures. The average absorbance at 650 nm (±standard error) for three technical replicates for each bioreactor is shown. Identical letters above bars indicate no significant difference among bioreactor means on a given day (*p* < 0.05).

**Figure 4.**
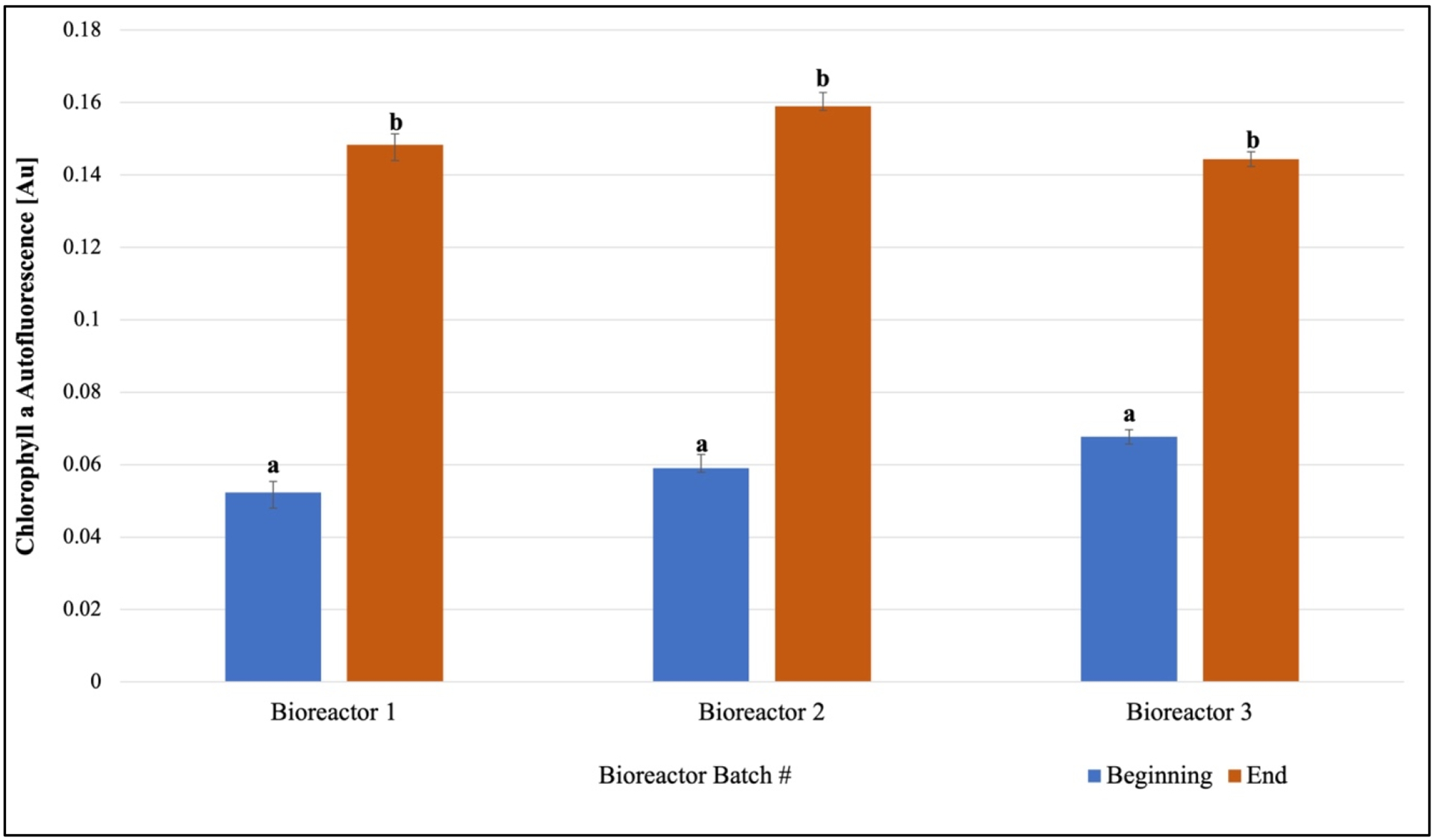
*Fremyella diplosiphon* increase in abundance of chlorophyll *a* over a 15-day period is shown above. The average absorbance at 470 nm (±standard error) for three biological replicates for each treatment is shown. Identical letters above bars indicate no significant difference among bioreactor means on a given day (*p* < 0.05).

Once consistent growth of *F. diplosiphon* was established across all volumes and conditions tested, further studies were performed in 15 L bioreactors outdoors. We observed a significant increase in phycocyanin and chlorophyll *a* levels on day 15 in 15 L bioreactors grown outdoors (Figures 3 and 4). In addition, quantification of chlorophyll *a* and phycocyanin revealed concentrations of 0.0011 ± 5.83 × 10^−5^ μg/μL and 7.051 ± 0.067 μg/μg chlorophyll *a*.

### Hydrothermal liquefaction and Products:

**Figure 5.**
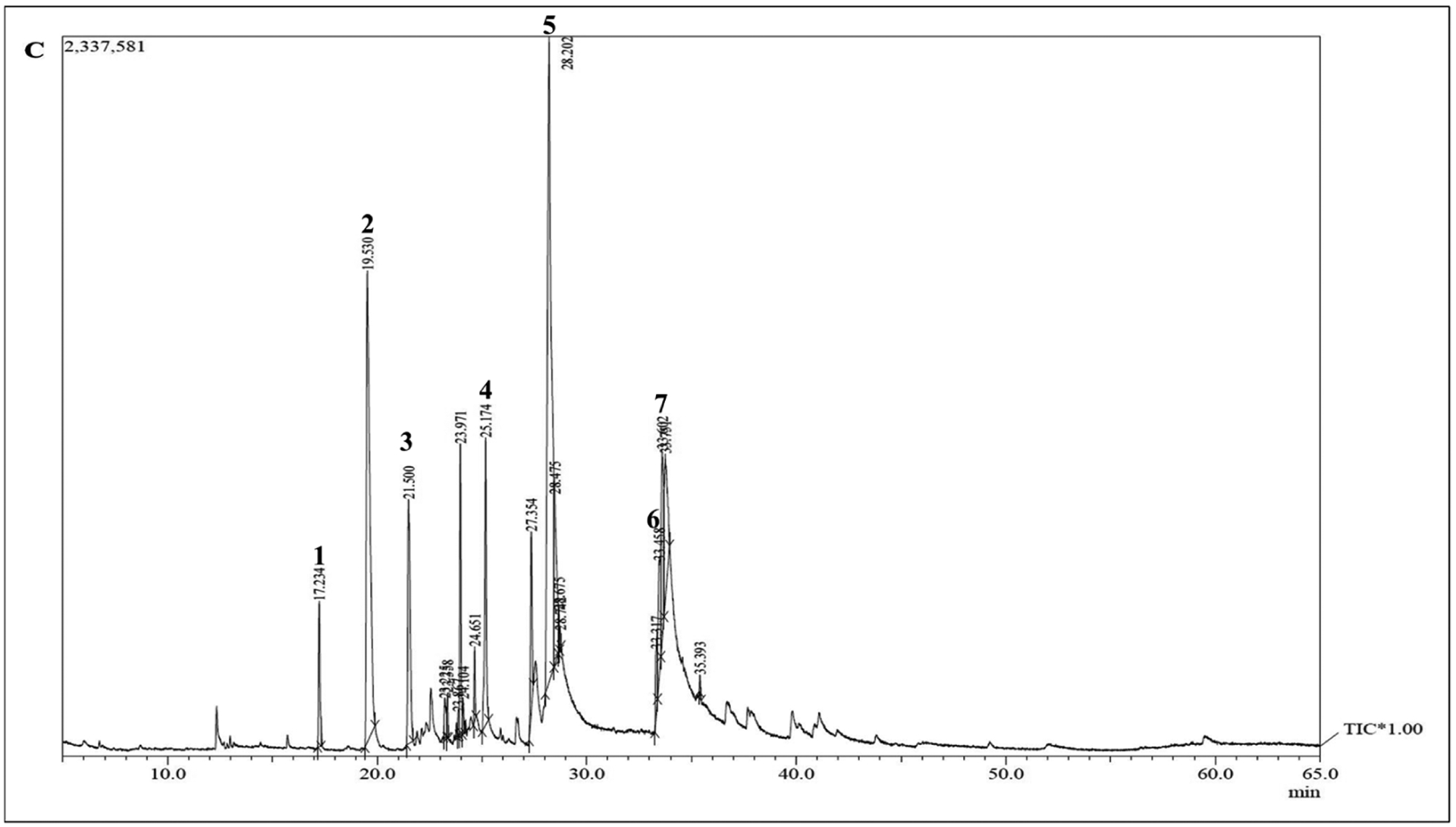
Gas chromatogram of hydrothermal liquefaction reactions from *Fremyella diplosiphon* SF33 biomass at 16 h heating intervals, which resulted in mostly complete reaction among the intervals tested. Numbers above peaks denote associated compounds in Table 1 below.

**Figure 6.**
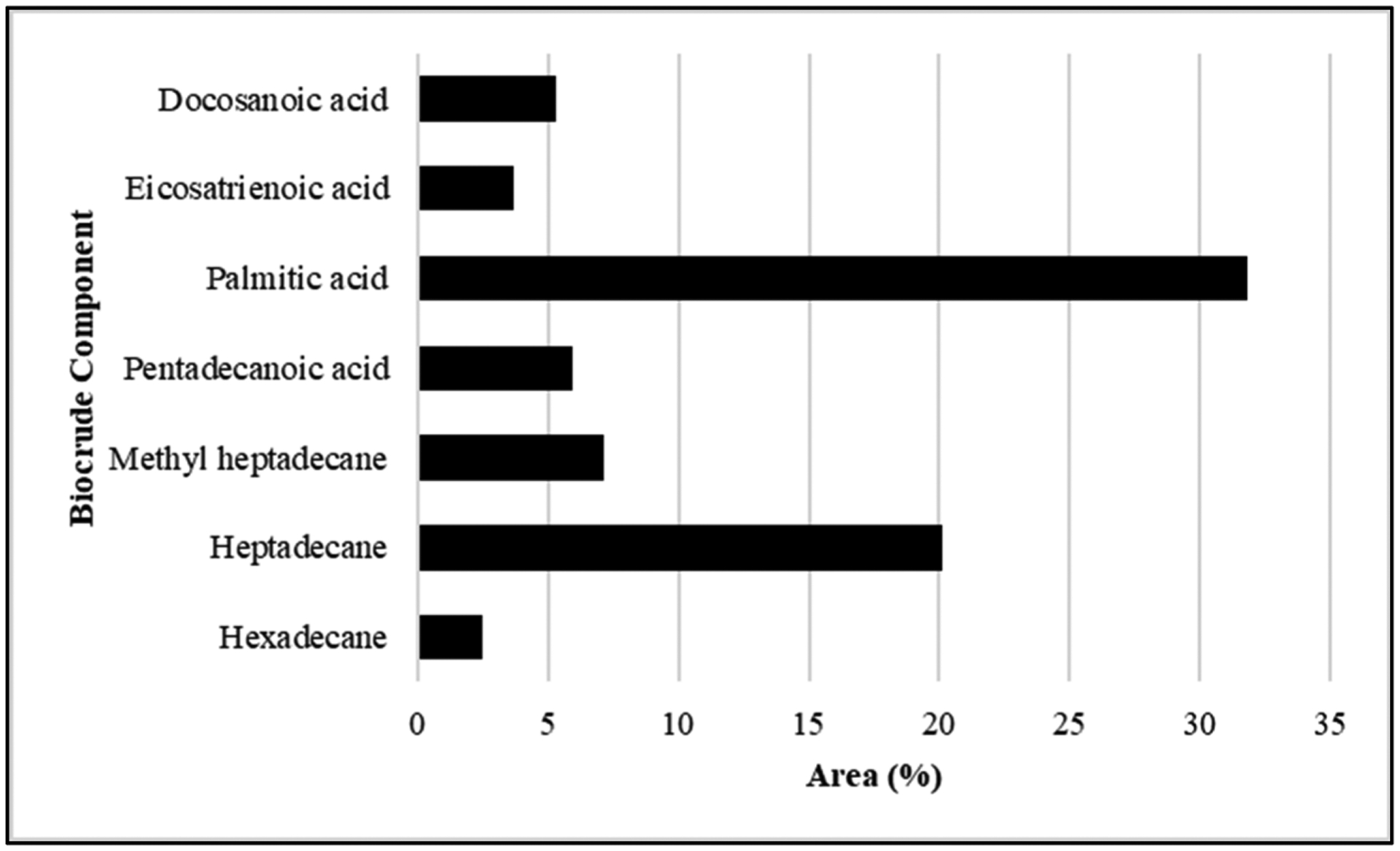
Breakdown of components identified and quantified in *Fremyella diplosiphon*-derived biocrude. Bars represent abundance of each component within the biocrude phase of hydrothermal liquefaction.

**Figure 7.**
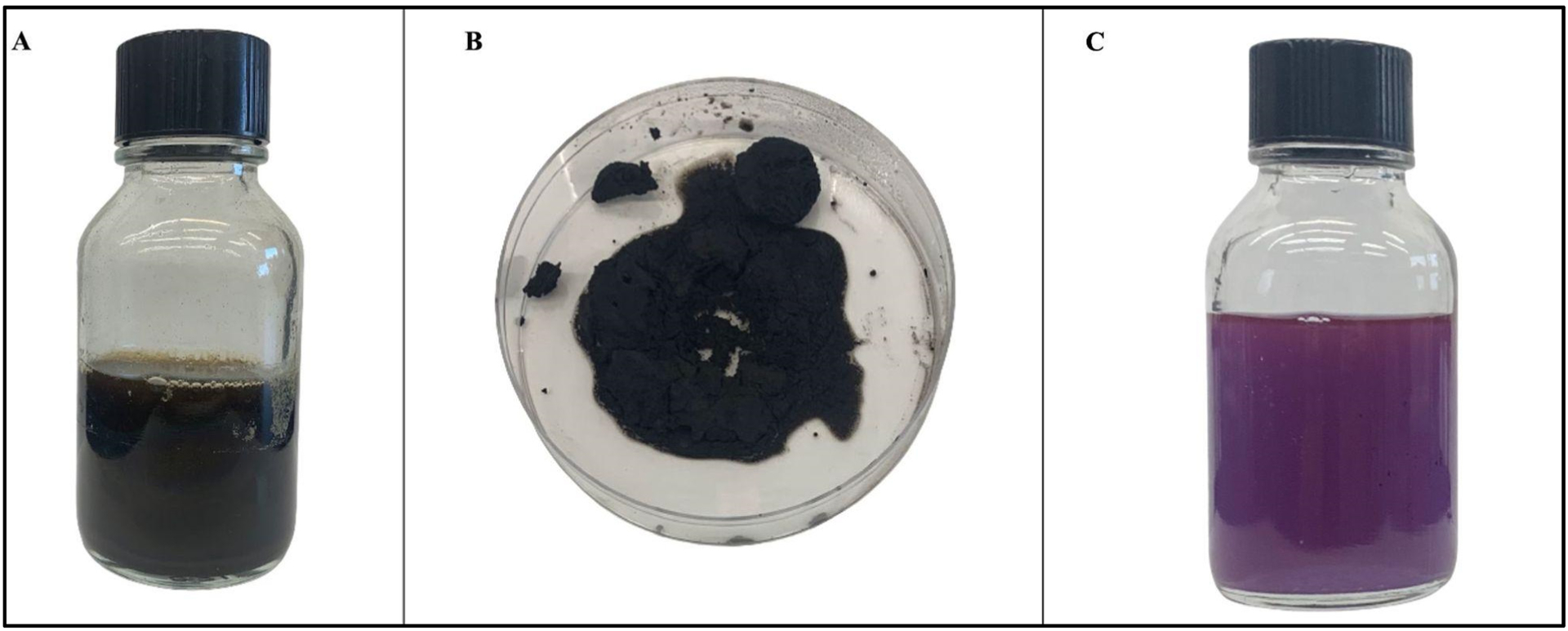
Products from hydrothermal liquefaction included (**A**) biocrude, (**B**) biochar, and (**C**) photosynthetic pigments from *Fremyella diplosiphon* biomass.

**Figure 8.**
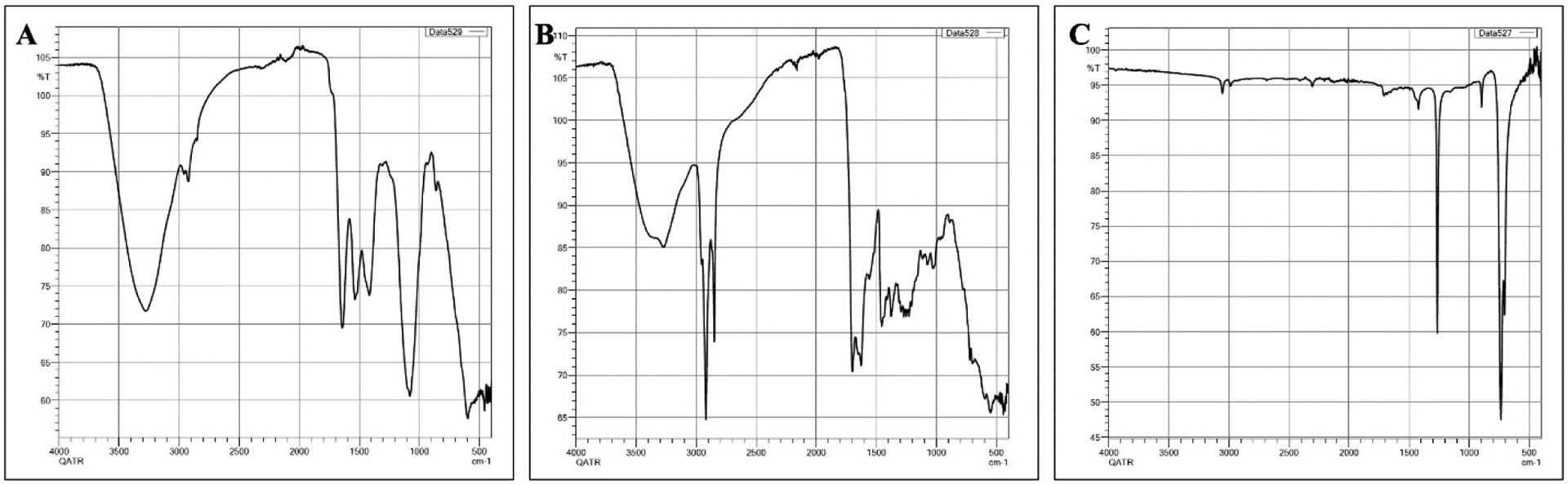
Fourier transform-infrared spectrum of *Fremyella diplosiphon*-derived (**A**) biomass, (**B**) biochar, and (**C**) biocrude.

Gas chromatography–mass spectrometry (GC–MS) of biocrude at different time intervals revealed that 16 h of heating during hydrothermal liquefaction resulted in the most complete reaction, with the least background noise (Figure 5). The HTL reaction resulted in dry weight yields of 1 g biocrude and 1.92 g biochar, a ratio of approximately 2 g biochar for every 1 g biocrude. GC-MS of the resultant product revealed components previously associated with biodiesel, including fatty acids, such as hexadecenoic (palmitic) acid and eicosatrienoic acid; alkanes, such as hexadecane and heptadecane; oxygenates, such as hexadecanol; methylated compounds, such as methyl heptadecane; and other compounds, such as aminohexanoic acid and 7-phenyl heptanoic acid (Table 1). We observed palmitic acid to be the most abundant component at 31.82% (Figure 6). Another co-product of the biocrude (Figure 7A) production process was biochar (Figure 7B), which is a lightweight, black residue composed of carbon and ash. In addition, photosynthetic pigments were isolated from cyanobacterial biomass (Figure 7C). While biocrude differed vastly from the biomass, as revealed using FTIR analysis, it was comparable to biochar, with just a few differences in the structure represented using known functional groups (Figure 8). Differences identified in the biochar included degradation of peaks near 1100, 1500, and 3400 cm^−1^, while peaks at 500 cm^−1^ were retained. In addition, peaks at approximately 1700 and 2900 cm^−1^ were observed in the biochar.

## 4. Discussion

Considering the current need for cost-effective biofuel in addition to value-added co-products, this study’s approach aimed to optimize and enhance *F. diplosiphon* biomass production in naturally available brackish water. An essential prerequisite for the large-scale cultivation of cyanobacteria for biofuel production is the identification of viable strains. As observed in this study, the high-performing strain has the potential for large-scale cultivation in raceway ponds or photobioreactors, and has a high lipid content enabling its use as efficient feedstock for biofuel production [11,20].

Greenhouse evaluation of *F. diplosiphon* SF33 indicated the ability of the strain to grow under fluctuating environmental conditions ranging from 13 to 32 °C. It should be noted that a drop in temperature to 5 °C on 11 April 2022, which was day 12 of the testing period in an experimental batch, did not hinder the survival of the strain. In addition, sporadic elevation of temperature in the greenhouse and outdoor-grown bioreactor cultures did not impact its survival, indicating the strain’s potential for commercial biofuel production. Contamination of large-scale cultures is a common problem that is encountered in scale-up of algal and cyanobacterial cultures. While we observed contamination in the fish tank cultures, which were grown in 10-gallon open aquariums, we were able to overcome this setback by increasing the initial culture OD_750_ from 0.1 to 0.2. This effort yielded consistent results in all the studies conducted, which suggests that this modification could eliminate potential contaminants.

Compounds identified from *F. diplosiphon*-derived biocrude have a wide range of commercial applications, such as the bioremediation of soil and water, thus enhancing the potential revenue from cyanobacterium. Biocrude was produced using hydrothermal liquefaction of wet *F. diplosiphon* biomass, suggesting that this is a suitable method for laboratory and small-pilot-scale cultivation. This accords with prior studies that produced biocrude from cyanobacteria and microalgae such as *Spirulina*, *Chlorella*, *Scenedesmus obliquus*, and *Botryococcus braunii* [13,21,22]. Biochar, another component of the hydrothermal liquefaction process, has various applications such as carbon capture, improving soil and water quality, and as animal feed [23]. Importantly, biochar improves water quality by removing cyanobacteria-derived toxins such as microcystins from harmful blooms [24]. Data from FTIR spectroscopy suggest the structural similarity of biochar to that of the total biomass; however, differences were identified in the functional groups. This is the first report of FTIR analysis in *F. diplosiphon*. Our results support the use of biochar for commercial applications, including the adsorption of heavy metal pollution [25]. FTIR analysis of biochar in the cyanobacterium *Spirulina* has been reported [26]. Using this as a model, alteration at 1100 cm^−1^, referring to C-O-C stretching, represents the breakdown of polysaccharides initially present in the biochar, while a peak at 3400 cm^−1^ depicts stretching of -OH in carboxylic acids or NH- stretching in a primary amine compound, indicating the presence of one or both of these substances in the biomass. Conversely, only biochar samples contained C=O and -CH bonds, suggesting the presence of carboxylic acids and aldehydes in this sample, which is in accordance with a report identifying these FT-IR peaks in the cyanobacterial strain *Synechocystis* PCC 6803 [27].

In addition to biocrude and biochar, other high-value co-products were identified in the scale-up process, thus increasing the potential commercial opportunities. A significant increase in phycocyanin and chlorophyll *a* abundance over a 15-day period indicated that scaling up cultivation did not have a detrimental effect on photosynthetic pigment accumulation. This is consistent with prior studies that have shown that pigmentation increases as growth increases; however, abiotic stresses such as salinity, light quantity, and nutrient availability affect chlorophyll *a* and phycobiliprotein levels [28–30]. This is logical since quantification of these pigments provides insight into photosynthetic efficiency, which directly impacts cellular macromolecules [31]. Phycocyanin, a pigment of great interest to the nutraceutical market due to its antioxidant properties quantified in the present study, offers a broad spectrum of commercial uses. Additionally, herbal retailers sell chlorophyll *a* for its immune and energy boosting effects. With a university start-up created, and the technology licensed (Appendix A), further scaling up of biomass production will be pursued at Morgan’s Patuxent Environmental and Aquatic Research Laboratory (PEARL). The strain offers a significant source of potassium, vitamins B1 and B2, niacin, and folate, and further efforts at bolstering its nutritional benefits will be explored (Intellectual Property Disclosure: 63/408,920).

## 5. Conclusions

Biofuels from cyanobacteria such as *F. diplosiphon* offer great value beyond their use as transportation fuels given their environmental benefits and lucrative co-products generated during fuel production. A significant reduction in greenhouse gas emissions through the blending of additives will further drive the commercial production and adoption of advanced biofuels. With the current initiatives undertaken, an era of using biofuels as an alternative to fossil fuels is on the horizon. Our results provide additional knowledge regarding scale-up cultivation, and the extraction and purification of bioproducts with real-world applications. Future studies will aim to use hydrothermal liquefaction as a scalable method for thermochemical conversion of cyanobacterial biomass, which will lead to the production of biocrude, as well as conduct a comprehensive analysis of fuel properties. This innovative research, which includes scaled-up cultivation systems and the development of a biofuel production system combining extraction and conversion to provide high biocrude yield, has great potential for commercialization.

## Supplementary Material

supplementary material

## Figures and Tables

**Table 1.** Selected biocrude-derived components in *Fremyella diplosiphon* and associated information, including type of molecule and known applications. Compounds were identified using similarity scoring with the National Institute of Standards and Technology (NIST) Mass Spectral Library database.

#	Component	Retention (min) Time	Application	Main Mass Fragment *(m/z)*
1	Hexadecane	17.234	Additive cetane number	70
2	Heptadecane	19.53	Additive cetane number, oxidative stability	57
3	Methyl heptadecane	21.5	Biological marker/standard	57
4	Pentadecanoic acid	25.174	Biodiesel precursor	82
5	Palmitic acid	28.202	Biodiesel precursor	73
6	Eicosatrienoic acid	33.458	Biodiesel precursor	79
7	Docosanoic acid	33.751	Biodiesel precursor	67

## Data Availability

The data presented in this study are available in this article and Supplementary Material.

## Appendix A. Patents

Inventions based on research efforts relating to *F. diplosiphon* have resulted in intellectual properties that have been licensed to one of Morgan State University’s first start-ups, HaloCyTech LLC. The company secured an exclusive license with MSU embodied in the following intellectual property: The halotolerant strain yielded U.S. Patent 10,626,363 entitled “Engineered cyanobacteria with Enhanced Salt Tolerance”. MSU has also filed a patent application entitled “Engineered Cyanobacteria with Enhanced Lipid Production” (U.S. Patent 10,793,883). In addition, surface plasmon resonance from gold nanoparticles was harnessed to enhance photosynthetic pigmentation and biomass accumulation in this species (“Composition and method for enhancing photosynthetic efficiency of microorganisms”, U.S. Patent 11,162,067). A non-provisional patent application for the use of iron nanoparticles has recently been filed in the USPTO (U.S. Patent Application 16/712,125). These patents can provide significant barriers to entry by blocking use (Table A1).

**Table A1.** United States Patent and Trademark Office information for the technologies licensed to HaloCyTech LLC.

Licensed Intellectual Properties from Morgan State University		
Issue #	Invention Title	Status
10,626,363	Engineered cyanobacteria with enhanced salt tolerance	Issued U.S. Patent
10,793,883	Engineered cyanobacteria with enhanced lipid production	Issued U.S. Patent
11,162,067	Composition and method for enhancing photosynthetic efficiency of microorganisms	Issued U.S. Patent
16*/*712,125	Iron nanoparticle-mediated enhancement of lipids in the cyanobacterium *Fremyella diplosiphon*	Utility Patent Application
17*/*891,482	Engineered cyanobacteria with enhanced UV tolerance	Non-provisional Application
63*/*408,920	Cyanobacteria as anti-inflammatory and nutritional feed	Provisional Application
