# Supplementary material for: Biocrude Production Using a Novel Cyanobacterium: Pilot-Scale Cultivation and Lipid Extraction via Hydrothermal Liquefaction
Source: Sustainability. Author manuscript; Available in PMC 2023 May 12. (PMC10181831; doi:10.3390/su15064878)
Supplement: supplementary material [file NIHMS1896871-supplement-supplementary_material.pdf]

Growth at Day 15

Descriptive statistics of your* k*=3 independent treatments:					
Treatment →	Bioreactor 1	Bioreactor 2	Bioreactor 3	Pooled Total	
Observations N	3	3	3	9	
Sum	1.66	1.682	1.689	5.031	
Mean	0.5533	0.5607	0.563	0.559	
Sum Of Squares	0.9187	0.9431	0.951	2.8127	
Sample Variance S	0.0001	0	0	0.0001	
Sample Std. Dev. S	0.0078	0.006	0.0053	0.0071	
Std. Dev. Of Mean	0.0045	0.0035	0.0031	0.0024	
One-Way ANOVA Of Your* K*=3 Independent Treatments:					
Source	sum of	degrees of	mean square	F statistic	p-value
	squares SS	freedom ν	MS		
Treatment	0.0002	2	0.0001	1.8369	0.2386
Error	0.0002	6	0		
Total	0.0004	8			
**Tukey HSD Results**					
Treatments	Tukey HSD	Tukey HSD	Tukey HSD		
Pair	Q statistic	p-value	inference		
B1 Vs B2	1.9704	0.4023924	insignificant		
B1 Vs B3	2.5973	0.2364134	insignificant		
B2 Vs B3	0.6269	0.8947191	insignificant		

Table S1: Growth of *Fremyella diplosiphon* SF-33 grown in 20 L bioreactors: Greenhouse 15 L culture on day 15.

Descriptive Statistics Of Your Kk=***3 Independent Treatments:***					
Treatment →	Bioreactor 1	Bioreactor 2	Bioreactor 3	Pooled Total	
Observations N	3	3	3	9	
Sum	1.5790	1.5700	1.5990	4.7480	
Mean	0.5263	0.5233	0.5330	0.5276	
Sum Of Squares	0.8311	0.8217	0.8524	2.5052	
Sample Variance Ss	0.0000	0.0001	0.0001	0.0000	
Sample Std. Dev. Ss	0.0040	0.0075	0.0072	0.0070	
Std. Dev. Of Mean	0.0023	0.0043	0.0042	0.0023	
One-Way ANOVA Of Your Kk=***3 Independent Treatments:***					
Source	sum of squares SS	degrees of freedom ν	mean square MS	F statistic	p-value
Treatment	0.0001	2.0000	0.0001	1.7674	0.2492
Error	0.0002	6.0000	0.0000		
Total	0.0004	8.0000			
**Tukey HSD Results**					
Treatments	Tukey HSD	Tukey HSD	Tukey HSD		
Pair	Q statistic	p-value	inference		
B1 Vs B2	0.8061	0.8279675	insignificant		
B1 Vs B3	1.7912	0.4627592	insignificant		
B2 Vs B3	2.5973	0.2364134	insignificant		

Table S2: Growth of *Fremyella diplosiphon* SF-33 grown in 20 L bioreactors: Outdoor 15 L culture on day 15.

Descriptive statistics of your k=*3 independent treatments:*					
	chlorophyll *a*				
Beginning	**Treatment**	Bioreactor 1	Bioreactor 2	Bioreactor 3	Pooled Total
	**Observations N**	3	3	3	10
	**Sum**	0.170	0.127	0.077	0.374
	**Mean**	0.043	0.042	0.026	0.037
	**Sum Of Squares**	0.009	0.006	0.002	0.018
	**Sample Variance S**	0.001	0.001	0.000	0.000
	**Sample Std. Dev. S**	0.025	0.021	0.013	0.020
	**Std. Dev. Of Mean**	0.012	0.012	0.008	0.006
End	**Observations N**	3	3	3	9
	**Sum**	0.033	0.036	0.060	0.129
	**Mean**	0.011	0.012	0.020	0.014
	**Sum Of Squares**	0.000	0.000	0.001	0.002
	**Sample Variance S**	0.000	0.000	0.000	0.000
	**Sample Std. Dev. S**	0.004	0.003	0.005	0.006
	**Std. Dev. Of Mean**	0.002	0.002	0.003	0.002

Beginning									
Source	sum of squares SS	degrees of freedom v	mean square MS	F statistic	p-value	treatments pair	Tukey HSD Q statistic	Tukey HSD p-value	Tukey HSD inference
Treatment	0.0012	2	0.0006	2.7311	0.1434	CB1 vs CB2	1.378	0.615	insignificant
Error	0.0014	6	0.0002			CB1 vs BC3	3.291	0.127	insignificant
Total	0.0026	8				BC2 vs CB2	1.913	0.421	insignificant
End									
Source	sum of squares SS	degrees of freedom v	mean square MS	F statistic	p-value	treatments pair	Tukey HSD Q statistic	Tukey I-ISD p-value	Tukey I-ISD inference
Treatment	0.0001	2	0.0001	4.38	0.0672	CB1 vs CB2	0.4243	0.9000	insignificant
Error	0.0001	6	0.0000			CB1 vs BC3	3.8184	0.0789	insignificant
Total	0.0002	8				BC2 vs CB2	3.3941	0.1156	insignificant

Table S3: *Fremyella diplosiphon* chlorophyll *a* quantification from 15 L bioreactor cultures at the beginning of culture and on day 15

Descriptive statistics of your k=***3 independent treatments:***					
	Phycocyanin				
Beginning	Treatment	Bioreactor 1	Bioreactor 2	Bioreactor 3	Pooled Total
	observations N	3	3	3	9
	sum	39338	42049	42773	124160
	mean	13113	14016	14258	13796
	sum of squares	516484254	589747445	610217821	1716449520
	sample variance s	329086	187322	187322	449168
	sample std. dev. s	574	433	433	670
	std. dev. of mean	331	250	250	223
End	observations N	3	3	3	9
	sum	62884	63253	63681	189818
	mean	20961	21084	21227	21091
	sum of squares	1318133670	1333766485	1351858913	4003759068
	sample variance s	592	59574	51163	41090
	sample std. dev. s	24	244	226	203
	std. dev. of mean	14	141	131	68

One-way ANOVA of your k=***3 independent treatments:***						**Tukey HSD results**			
Beginning									
source	sum of squares SS	degrees of freedom v	mean square MS	F statistic	p-value	Treatments pair	Tukey HSD Q statistic	Tukey HSD p-value	Tukey HSD inference
Treatment	2,185,880	2.0	1,092,940	4.6592	0.0601	B1 vs B2	3.2317	0.134	insignificant
Error	1,407,462	6.0	234,577			B1 vs B3	4.0947	0.062	insignificant
Total	3,593,342	8.0				B2 vs B3	0.863	0.807	insignificant
End									
source	sum of	degrees of	mean square	F statistic	p-value	treatments pair	Tukey HSD Q statistic	Tukey HSD p-value	Tukey HSD inference
	squares SS	freedom v	MS			B1 vs B2	1.1059	0.716	insignificant
treatment	106,061	2	53,030	1.429	0.3108	B1 vs B3	2.3887	0.284	insignificant
error	222,659	6	37,109			B2 vs B3	1.2827	0.650	insignificant
total	328,720.8	8							

Table S4: *Fremyella diplosiphon* phycobiliprotein phycocyanin quantification from 15 L bioreactor cultures at the beginning of culture and on day 15.
